# A Data‐Driven Approach Identifies Subtypes of Caries From Dental Charting

**DOI:** 10.1111/cdoe.13014

**Published:** 2024-10-22

**Authors:** Simon Haworth, Lisa Kastenbom, Peter Persson, Niklas Fries, Anders Esberg, Daniel Jönsson, Ingegerd Johansson

**Affiliations:** ^1^ Bristol Dental School University of Bristol Bristol UK; ^2^ Department of Odontology Umeå University Umeå Sweden; ^3^ Department of Clinical Sciences Lund University Malmö Sweden; ^4^ Public Dental Service of Skåne Lund Sweden; ^5^ Faculty of Odontology Malmö University Malmö Sweden

**Keywords:** caries, dental register, latent class analysis, phenotype‐wide association study, Sweden

## Abstract

**Objectives:**

The objectives were to: (i) assess the accuracy of dental data for adults obtained from the Swedish Quality Register on Caries and Periodontitis (SKaPa); (ii) explore whether Latent Class Analysis (LCA) can identify groups of people based on caries data; and (iii) characterise the dental, medical and behavioural characteristics of people in the LCA‐derived classes.

**Methods:**

Caries data from the SKaPa register were compared with clinical data collected by five experienced dentists in a nested subgroup of the Malmö Offspring Study (MOS), namely the Malmö Offspring Dental Study (MODS) (*n* = 724) for validation. Dental data from SKaPa were then used to classify 61 984 adult participants of the Västerbotten Intervention Programme (VIP) into five classes using LCA and DMFS‐based quintile ranking, respectively. Dental status (including caries progression over 5 years), medical, anthropometric and behavioural characteristics were compared between the groups. Analyses were replicated in 2767 adults in the MOS.

**Results:**

DMFS‐scores and number of teeth recorded within −2 to +2 years showed excellent agreement between the SKaPa and reference data with intra‐class correlations > 0.90. The five LCA classes differed in mean DMFS from 10.0 to 94.4. There were strong associations between LCA class and health, and health and behavioural measures respectively, including some associations that were not detected using DMFS‐ranked quintile groups. LCA class was associated with incremental change in DMFS, DFS, and number of teeth. The results in the MOS cohort were consistent with the results in the VIP cohort.

**Conclusions:**

Dental data for adults from the SKaPa registry were considered accurate within 2 years of recording. The LCA approach can classify participants into caries subtypes based on dental charting. These groups differ in health and behavioural characteristics and future caries increment. The LCA approach may capture some information that is missing from DMFS‐ranked quintile groups, but is also heavily influenced by total DMFS, meaning that applying LCA in cumulative, highly age‐determined diseases, such as caries, is a challenge.

## 1 Introduction

Complex diseases are influenced by a range of underlying susceptibility and protective factors such as host genetics, social and behavioural characteristics and treatment. The interplay between these factors may contribute towards variation in the clinical presentation of disease, and may give rise to the existence of disease subtypes. There is growing interest in searching for these subtypes in complex disorders including cardiometabolic diseases, cancer, depression and Alzheimer's disease [1, 2, 3, 4]. The ability to identify groups of people with severe or high risk subtypes of disease is potentially useful in a range of scenarios including targeted public health strategies and routine clinical care, as well as helping to provide more personalised and specific counselling and treatment under the precision medicine concept [5].

Dental caries is among the most prevalent, treatment‐demanding, yet preventable, diseases worldwide [6]. It arises from complex interactions of genetic, biological, behavioural, and environmental factors, where dietary intake of free sugars is a key component [7], but is also recognised as a disease of social deprivation [8]. It presents with substantial variation in clinical manifestation. Despite counselling on oral hygiene, dietary risk factors and fluoride use, 15%–20% of children and adults remain with significant disease activity even in high‐income countries [9]. There is evidence from data‐driven hierarchical clustering (HCA) of tooth surface status showing that tooth surfaces fall into clusters [10] and these are influenced by different genetic factors [11], suggesting different underlying biology. Similarly, latent class analysis (LCA) has identified subgroups of children with early childhood caries, revealing distinct patterns of disease and tooth microbiota profiles [12], as well as differing caries trajectories [13]. Collectively, the complexity of the upstream determinants of caries as well as the available data from children and genetic investigations suggest that the conditions exist under which there are likely to be different subtypes of caries in adults. To date, there has been little work applying LCA to investigate subtypes of caries in adults, and it is unclear whether LCA‐classification of caries signs can be used to identify meaningful caries subtypes. This study aimed to investigate this by applying a data‐driven approach to identify subgroups of caries from dental charting in an adult population.

A necessary prerequisite for this is valid dental charting in a large adult population. Adoption of electronic charting in dental practices provides a potential solution to this problem. In Sweden, the Swedish Quality Register on Caries and Periodontitis (SKaPa, www.Skapareg.se) is a comprehensive dental register for both children and adults [14]. Personal identification numbers allow linkage of SKaPa‐data to other medical and demographic registers, or population‐based cohorts with screening data and biological samples, such as the Västerbotten Intervention Programme (VIP) [15] and the Malmö Offspring Study (MOS) [16]. While the validity of caries data in SKaPa has been confirmed for children aged 6 and 12 years [17], its accuracy in adults remains unevaluated. This is needed as the use of SKaPa data in studies pertaining to caries risk prediction and its association with other diseases is on the rise, both within Sweden and internationally.

The study (i) evaluated the validity of dental data in adults obtained from the Swedish Quality Register on Caries and Periodontitis (SKaPa); (ii) explored whether latent classes can be identified based on caries information derived from SKaPa; and (iii) characterised dental, medical and behavioural characteristics, including longitudinal change in caries status in young adults to younger elderly, in the LCA‐derived classes.

## 2 Methods

### 2.1 Study Populations

The study encompassed two populations in Sweden (the VIP [15], and the MOS [16]). Beginning in 1986, inhabitants of Västerbotten County in northern Sweden were invited to a health screening when they reached the age of 30 (though this age group was only included for a few years), 40, 50, and 60. Consenting participants were subsequently enrolled in the VIP cohort [15]. As of December 2021, approximately 133 000 individuals had provided questionnaire data, with 50% undergoing two or more repeat screenings. For the present study, caries status was searched in the SKaPa register [14]. The results from VIP were externally validated using data from the MOS cohort, located in southern Sweden [16]. The MOS, conducted from 2013 to 2021, includes children and grandchildren of participants from the Malmö Diet and Cancer Study (MDC) [18]. For both cohorts, participants with dental data in SKaPa and aged 18 through 69 years were included. Additionally, the validity of caries information from the SKaPa register was assessed in a dental substudy within the MOS (MODS).

The project adheres to the Helsinki Declaration and *General Data Protection Regulation* (*GDPR*) including that all participants gave written consent when recruited to the basic cohorts and was approved by the Swedish Ethical Review Authority Dnr 2020–01416 and Dnr 2020–06560 and MOS 2012/594, MODS 2013/560.

### 2.2 Dental Caries Status

Caries status at the tooth surface level was searched for in the SKaPa register using personal registration numbers. Dental data originated from examinations by dentists or dental hygienists in public or private dental offices in the Västerbotten (VIP) and Skåne (MOS) regions in northern and southern Sweden, respectively. For the incisor teeth, 4 surfaces were scored, and for premolar and molar teeth, 5 surfaces. Third‐molar teeth were excluded. The caries scores were defined as: D0 for untreated and clinically sound tooth surfaces, D1 for caries in the outer enamel, D2 for caries extending into the enamel‐dentin border, and D3 for caries in the dentine. Surfaces with a fissure sealant, enamel hypoplasia, fluorosis, or tooth wear were recorded as D0 and restored surfaces as D3. For missing or crown‐covered incisor 4 surfaces were scored as D3 and for premolars and molars 5 surfaces. Non‐erupted and congenitally missing teeth were imputed as caries‐free. A tooth surface was considered as caries affected if assigned as a D2 or a D3 and the sum of caries affected surfaces was aggregated to the DMFS (including decayed, missing, and filled surfaces) and DFS (including decayed, and filled surfaces) indexes.

A total of 1030 adults enrolled in the MODS substudy, of whom 1024 completed a dental examination. Caries status was recorded by visual inspection, probing using a double‐ended dental explorer (Hu‐Friedy EXD57) and bite‐wing radiographs. Of these, 724 individuals (71%) had a match in the SKaPa register and were used for validation, with the SKaPa data serving as the test method and the clinical examination data serving as the reference method.

### 2.3 Behavioural Characteristics, Anthropometric and Medical Data

For the participants in the VIP cohort, questionnaire data provided information on behavioural characteristics (smoking, snus use) and highest educational level. Questionnaire data was supplemented by anthropometric measurements (BMI [weight/height^2^], wdaist circumference) and laboratory tests (triglycerides, total and HDL cholesterol, fasting blood glucose, 2‐h post‐glucose challenge levels, systolic and diastolic blood pressure) from clinical assessments [15]. Implausible values for anthropometric or laboratory measures, as defined by Region Västerbotten (https://www.umu.se/enheten‐for‐biobanksforskning/provsamlingar‐och‐register/northern‐sweden‐health‐and‐disease‐study) were excluded. Information on dietary habits, including intake of 66 foods/food groups and estimated energy (kCal/day), macronutrients, and alcohol was available from a food frequency questionnaire (FFQ). A slightly longer version of the FFQ has been validated against repeated 24‐h recalls [19] while the 66‐item version used in this study has been validated against biomarkers for B vitamins [20]. A subset of the corresponding data was available for MOS.

### 2.4 Data Handling and Statistical Analyses

Number of teeth and aggregated caries scores (DMFS) derived from SKaPa (test data) and the reference scoring were evaluated by Spearman correlation coefficients, intra‐class correlation coefficient (ICC, using a two‐way mixed model with an agreement coefficient and single measures) between the test and reference groups and by comparing mean DMFS values in quartiles from the DMFS distributions. Due to a bimodal age distribution in the MOS, these analyses were conducted separately for participants < 40 years and ≥ 40 years (see Figure 2B).

Latent Class Analysis (LCA) was applied to explore hidden structures in the caries pattern across the 128 tooth surfaces using the poLCA package in R. A series of models with 1–9 classes were run for all participants, to identify a suitable number of classes. Model selection was based on the Akaike information criterion (AIC), Bayesian Information Criterion (BIC), and entropy values; ultimately, a five‐class model was chosen where the AIC and BIC values plateaued at a low level (Figure S1). Subsequently, five‐class LCA models were run based on the first available dental recording including sex and age as covariates. LCA‐derived classes are non‐ordinal however, for ease of presentation, the class numbers were recoded to represent the lowest (code I) to the highest (code V) based on mean DMFS. For comparison, participants were also ranked into quintiles (by sex and 10‐year age group, coded Q1–Q5) based on the DMFS distribution.

Dental status and phenotypical characteristics were compared across LCA and quintile groups, respectively. Categorical data are presented as numbers or percentages. Continuous data are presented as means with standard deviations (SD). Group differences were tested in ANOVA in generalised linear modelling (GLM) with sex as covariate. Medical and behavioural characteristics were compared for participants with a VIP visit within 2 years apart from the dental recording and for MOS participants from the same year. Triglyceride measures were logarithmically transformed, and energy‐providing nutrients and alcohol intake were evaluated as intakes of grams per day and their contribution to total energy intake (E%).

In the five LCA and quintile classes, incident change in dental status was examined in participants with baseline and 5‐year follow‐up visits (*n* = 42 540 in VIP and *n* = 1764 in MOS). The analyses were restricted to participants with a reported DMFS difference ≥ 0. Tests for difference in 5‐year incidence between groups were carried out using generalised linear modelling. The ability of LCA‐group and quintile group to predict incident change was compared using AIC and a relative likelihood test was used to test the null hypothesis that the LCA approach was non‐superior. Corresponding criteria and analyses were applied to the MOS data.

## 3 Results

### 3.1 Study Numbers and Group Characteristics

**FIGURE 1:**
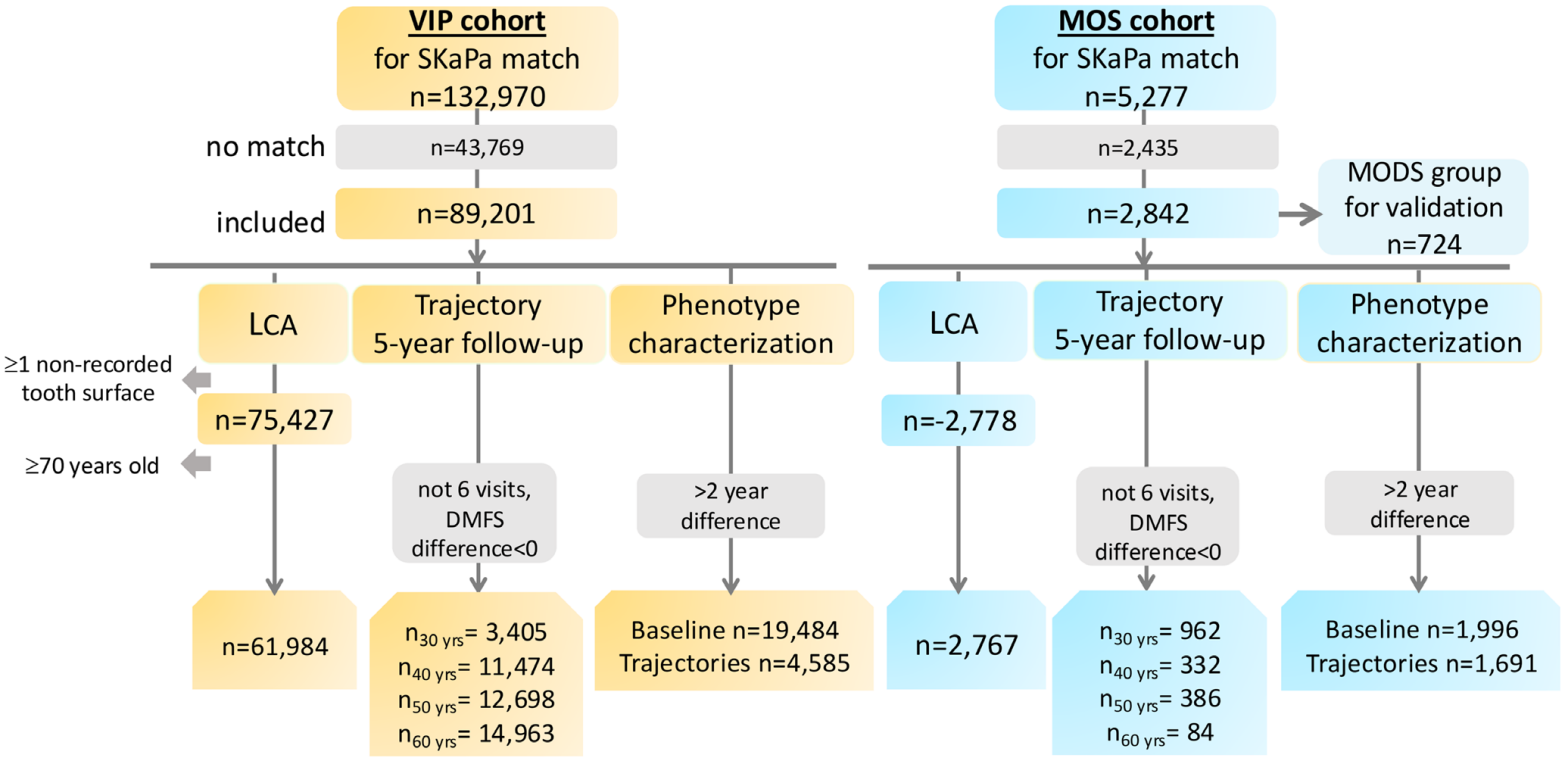
CONSORT diagram showing the number of eligible participants and their inclusion in different analyses for the VIP and MOS cohorts. Grey boxes indicate exclusion criteria, such as ‘> 2‐year difference’ meaning that participants with time spam between dental and medical screenings exceeding 2 years were excluded.

For VIP, dental records were sought for 132 970 individuals, with a match for 89 211 individuals (67.1%), of whom 10 lacked information on caries status at the tooth surface level (Figure 1). Of these, 27 217 participants were excluded due to being 70 years or older, or because status for single teeth was not reported, leaving 61 984 participants for the downstream analyses. In the MOS cohort, records were sought for 5277 participants, with 2842 (53.9%) matches for caries, and 75 were excluded for the reasons mentioned above leaving 2767 participants for analyses (Figure 1).

**FIGURE 2:**
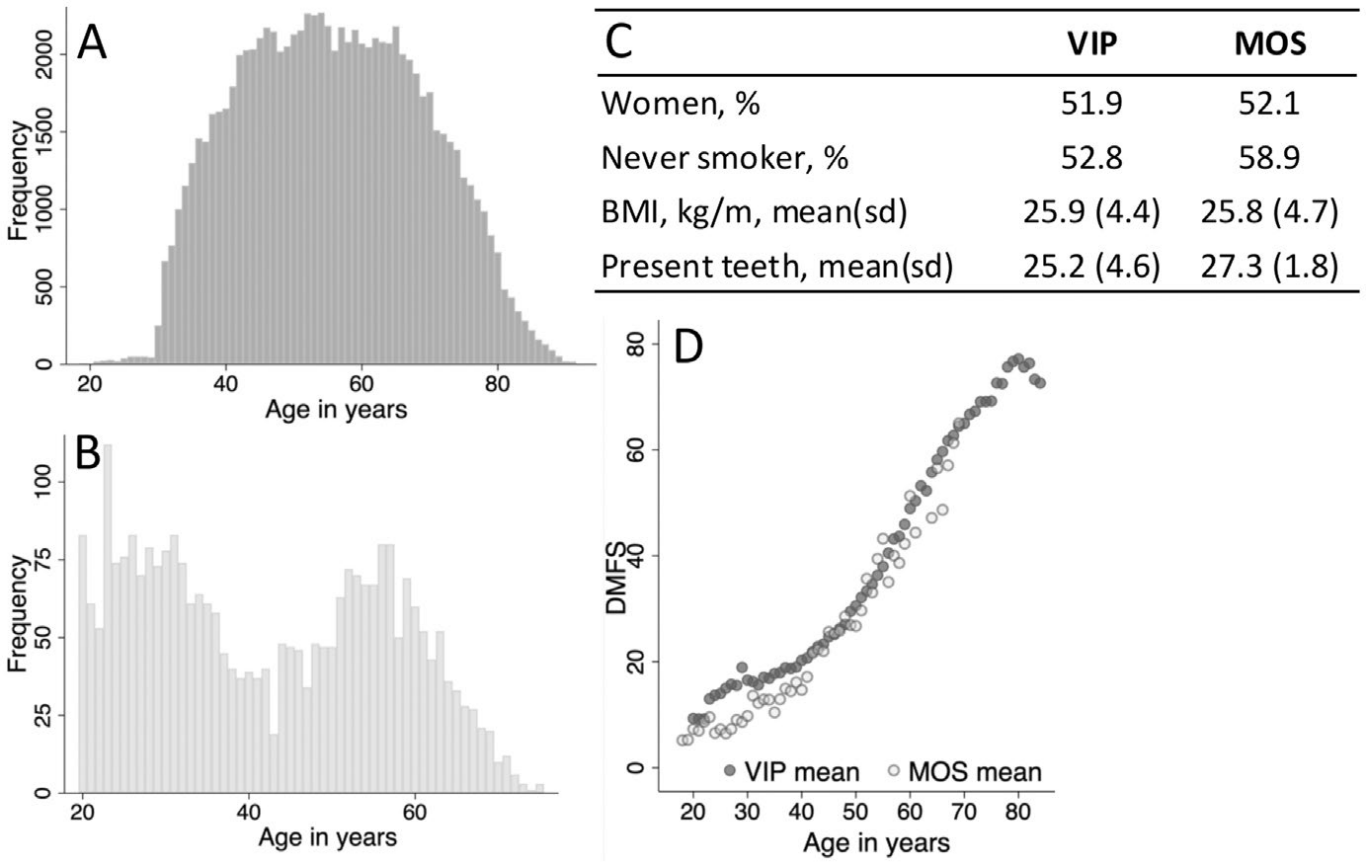
Age distribution in the (A) VIP and (B) MOS cohorts, (C) some demographic characteristics of the two cohorts and (D) cross‐sectional mean DMFS by age.

The distribution of age was approximately normal in VIP, but because MOS invited the children and grandchildren of MDC participants the age distribution was bimodal in MOS (Figure 2A,B). Both cohorts had similar proportions by sex, similar proportions of never‐smokers, and similar mean BMI (Figure 2C). DMFS was strongly associated with age in both cohorts with similar age patterns (Figure 2D).

### 3.2 Validity of SKaPa‐Derived Caries Information

**FIGURE 3:**
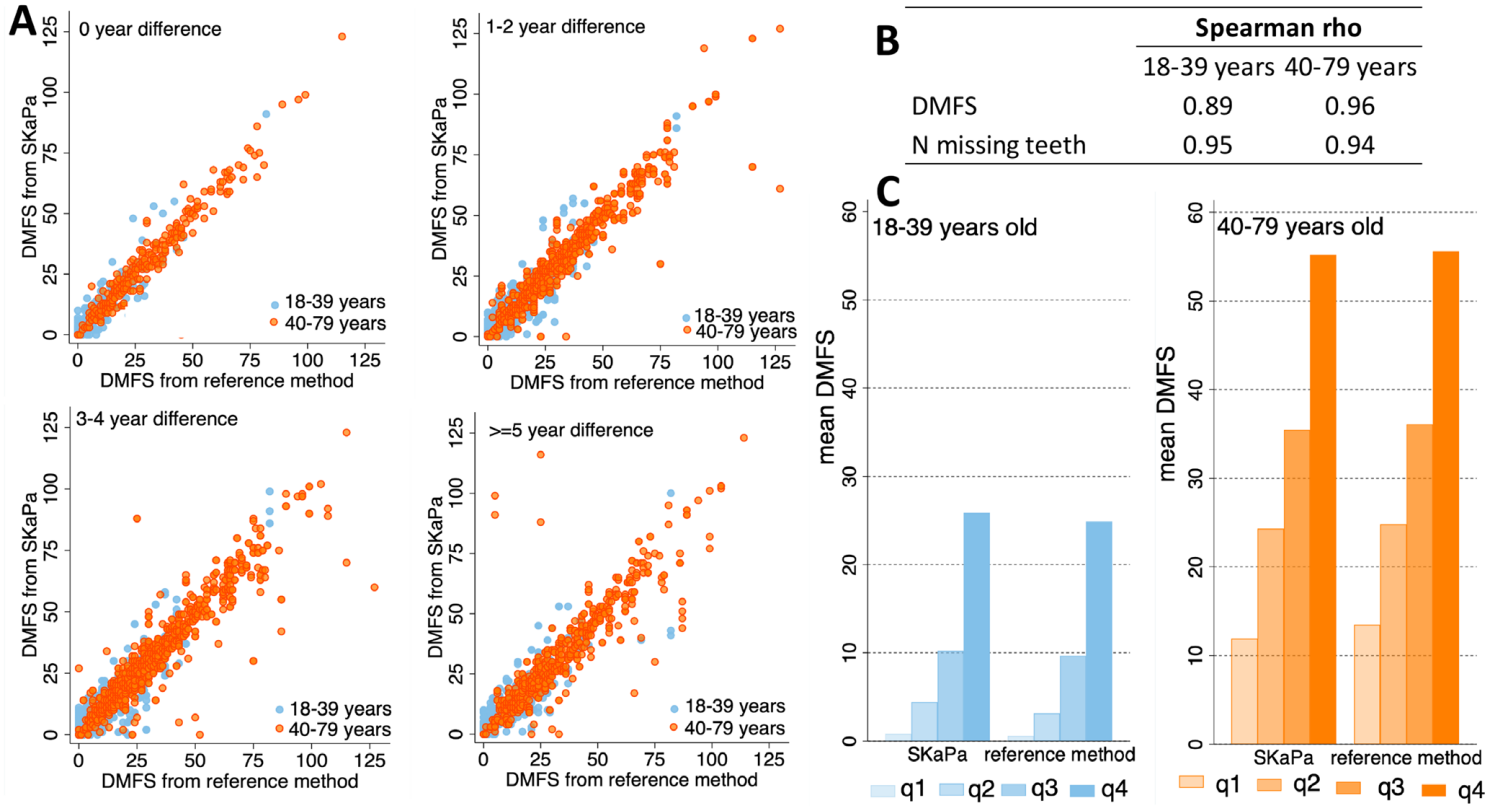
(A) Scatter plots for DMFS assessed in the reference examination versus those from SKaPa for those < 40 years (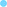) and those ≥ 40 years (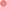) and by (absolute) number of years between the SKaPa and reference examinations; (B) Spearman correlation coefficient between SKaPa and reference DMFS scored the same year; (C) mean DMFS values in quartile groups ranked by sex and 10‐year age group classifications from SKaPa and reference caries distributions, respectively.

Of 1024 MODS participants in the dental substudy, 724 (71%) had SKaPa information available (Figure 1). The sex and age distribution among these participants mirrored that of the larger MOS cohort, that is half being women and half men, and half below and half above 40 years of age. Scatter plots of reference data versus SKaPa‐derived DMFS‐values showed a virtually linear relation for data from the same year (Figure 3A upper left plot), and when including data spanning from −2 to +2 years (Figure 3A upper right plot), and with ICCs > 0.90. Time difference beyond 2 years increased discrepancies between the two methods, and ICCs fell below 0.90 (Figure 3A lower panels). In agreement, high (≥ 0.89) Spearman correlation coefficients were seen for DMFS and number of teeth assessed by the two methods up to 2 years apart (Figure 3B), and mean scores in quartile‐ranked groups from SKaPa and reference DMFS distributions, respectively, did not differ (Figure 3C).

### 3.3 Characteristics of LCA Identified Classes Versus DMFS Ranked Quintiles

**FIGURE 4:**
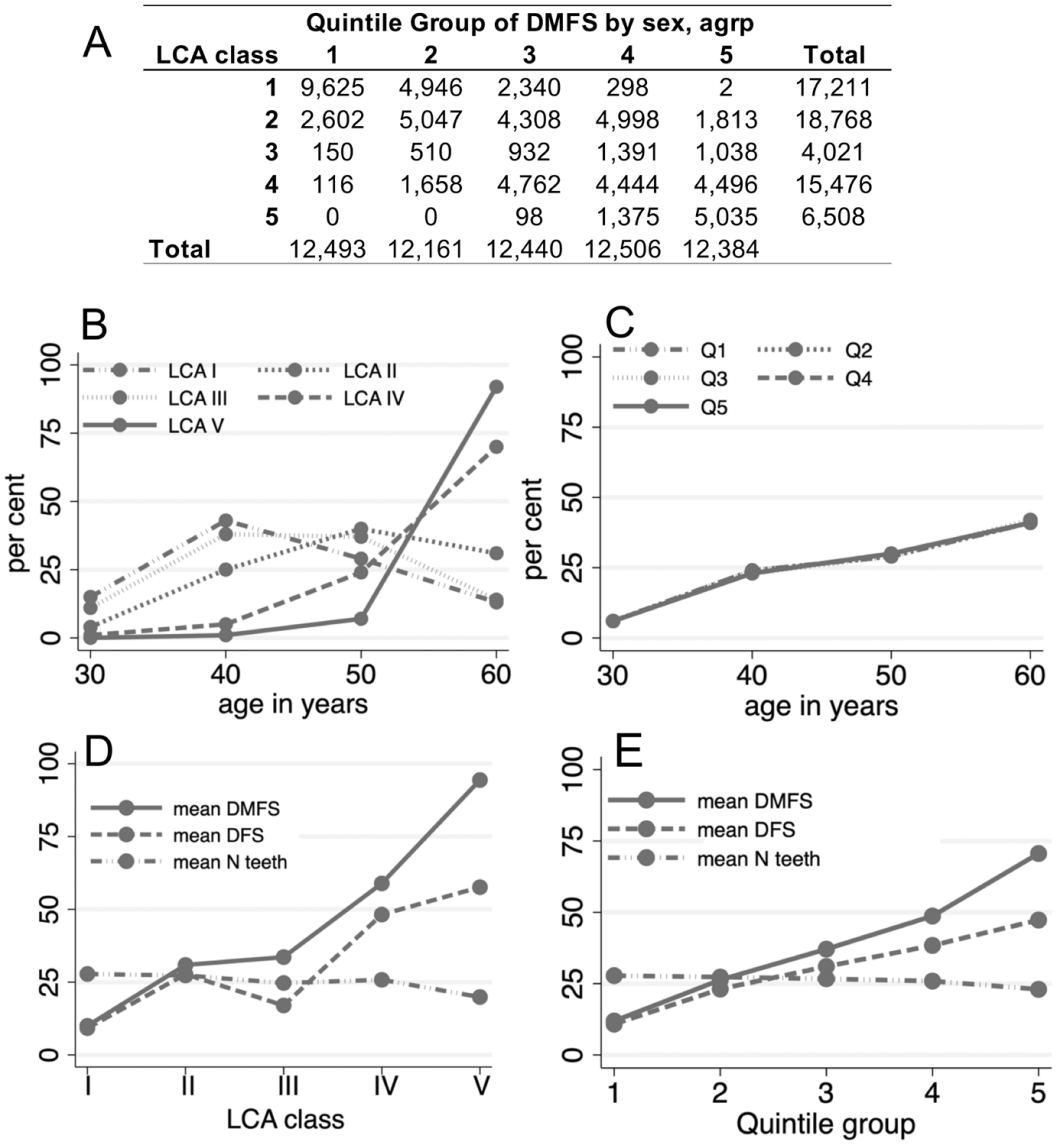
(A) Participant cross‐tabulation between classification into five LCA classes and quintile ranking groups derived from sex and 10‐year age group DMFS distribution. (B, C) Age distribution for (B) LCA classes and (C) DMFS quintile groups across all participants with a baseline visit (*n* = 61 984). The five lines in (C) are nearly superimposed following ranking in sex and age‐group strata. (D, E) Mean DMFS, DFS and number of present teeth across (D) the LCA classes and (E) the DMFS quintile groups.

The size of the LCA classes varied, with Class II including 18 768 participants and Class III 4021 participants (Table S1). This contrasted with the more uniform numbers observed in quintile groups derived from DMFS ranking. Notably, there was some overlap in participants classified into either the lowest or highest quintiles of LCA and DMFS groups (Figure 4A). The distribution of participants across four 10‐year age groups showed variation by LCA class, with a notable predominance of the oldest group in LCA classes IV and V despite attempting to account for sex and age as covariates in the models (Figure 4B). A similar pattern between age and DMFS‐ranked quintile groups was also seen (Figure 4C). Mean DMFS scores varied across LCA classes, from 10.0 in Class I to an extremely high 94.4 in Class V (Figure 4D), while the range across quintile groups was from 11.9 to 70.6 (Figure 4E). The rise in DMFS scores was accompanied by a continuous reduction in the number of teeth and, except for LCA Class III, an increase in DFS‐scores (Figure 4D,E). Although the sex distribution differed among the classes and quintiles, no consistent trend was evident across these groups (Table S1).

**FIGURE 5:**
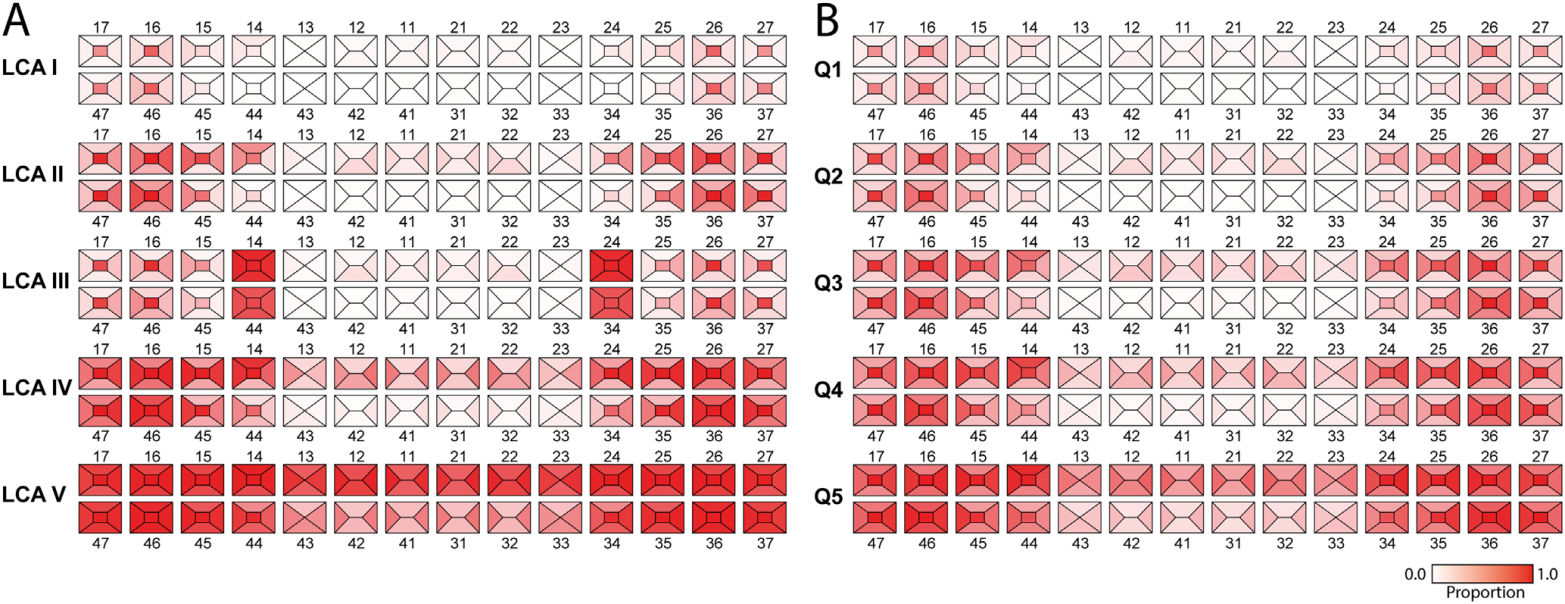
Panel of odontograms illustrating mean caries prevalence per tooth surface for (A) the five LCA classes (class I–V) and (B) the five quintiles based on the DMFS distribution in VIP (Q1–Q5) in the VIP cohort. White indicates caries‐free (0), and dark red that all surfaces are caries‐affected (1).

Alongside differences in total caries experience, there were distinct shifts in the spatial distribution of caries‐affected surfaces when comparing LCA and quintile groups. Thus, LCA class I was defined by affected occlusal surfaces in molar regions and class II by all surfaces in the molar regions and second premolars. In LCA class III ‘caries’‐scores were concentrated on the first premolars, while the pattern of class IV appeared similar to class II but with higher mean prevalence per tooth surface. Finally, class V participants had very high caries experience in all regions, including lower incisors (Figure 5A). The pattern for the DMFS quintiles was similar overall, although the pattern of affected surfaces differed between Q3 and LCA class III, and the overall caries prevalence in Q5 was less extreme than in LCA class V (Figure 5A vs. Figure 5B).

LCA class was associated with risk markers for complex diseases, i.e., smoking, BMI, waist circumference, plasma triglycerides, blood sugar levels, and intake of total carbohydrates and sucrose (Table S1A). Many of these associations were also observed between risk markers and quintile group (Table S1B), with generally poorer model fit statistics suggesting the LCA approach generally performs better at identifying groups with distinct health and behavioural characteristics. There were some associations which were only detected using one approach. As an example, association between 2‐h blood sugar following a glucose challenge was seen with LCA class (*p* = 1.8 × 10^−39^) but not with quintile group (*p* = 0.052).

### 3.4 Latent Class as a Predictor of Caries Increment

The change in DMFS was estimated in the subset of 42 540 VIP participants with a baseline and at least five follow‐up visits (Table S2). There was a U‐shaped relationship between LCA class and DMFS increment, with the fastest rate of change in LCA class V and the slowest increment in LCA class III. A similar U‐shaped relationship was seen between DMFS quintile and caries increment, with the slowest increment in Q2 and Q3. LCA group V and quintile group Q5 had the highest rates of tooth loss and lowest rates of DFS increment during follow‐up.

### 3.5 Replication in the MOS Cohort

LCA classification was carried out de‐novo (i.e., with no prior model knowledge from VIP) among 2767 MOS participants using VIP‐filtering conditions. Baseline and 5‐year follow‐up results in MOS largely mirrored those seen in VIP (Tables S3 and S4). Thus, LCA classes I and II were large groups, and class III was the smallest with health and behavioural characteristics and number of remaining teeth deviating from the other classes, and the highest prevalence in first premolars of a condition rated D3 (Figure S2). The correlation between the LCA class and DMFS‐ranked quintiles in MOS was consistent with the patterns seen in VIP. LCA class was associated more strongly than quintile group with BMI, and smoking, and LCA class III exhibited the lowest increase in DMFS scores over a period of 5 years, matching what was seen in VIP (Table S4).

## 4 Discussion

The study tested and confirmed the validity of caries data in a national register and used this in an LCA approach to search for subtypes of caries from dental charting in a large cohort in northern Sweden. Five classes were found, which replicates in an independent cohort in southern Sweden, and who differed in dental caries status, medical and behavioural characteristics but also age. Reflecting this age‐driven effect, the LCA classes were strongly correlated with DMFS‐based ranking groups for most, but not all classes.

The five subtypes identified in the study had similar proportions and characteristics in the discovery and replication datasets. In cross‐sectional analysis, these LCA groups were associated with health and behavioural traits with generally stronger association patterns than those observed using DMFS‐ranked quintile groups. Class III emerged as a group with distinct distribution of caries‐affected surfaces (with D3‐scored surfaces enriched for first premolars), better medical and behavioural profile than nearby classes and low caries increment during follow‐up. This class may represent a group where first premolars have been extracted for orthodontic reasons. Class V emerged as a group with high caries experience at baseline, high social and behavioural risk factors and high DMFS increment and tooth loss during follow‐up. Class V had low DFS increment during follow‐up, which can be interpreted as a ceiling effect [21], given this group has few sound tooth surfaces available to develop new caries, similar to the effect previously reported in children [13].

The classification system used here was based on dental charting and could potentially be applied in healthcare systems without costly biomarkers or additional examinations. One potential use case would be as an empirical way to identify groups of high risk people who may benefit from targeted intervention. The design of such an intervention is not considered in the present study, but is suggested as a topic for future research. There are natural limitations to public health interventions which only target high risk groups, since the majority of the population receive no benefit, and universal interventions tend to be most cost‐effective [22]. If the LCA groups are not useful for public health and clinical practice, they may still be helpful to conceptualise caries in epidemiological studies. Specifically, the LCA groups tended to have stronger associations with health and behavioural characteristics compared to quintile groups, and subtleties in associations such as the U‐shaped association between subgroup and DMFS increment would not be visible in analysis using DMFS.

One challenge with applying LCA in adult populations is the highly age‐determined distribution of DMFS, which naturally tends to create groups of older versus younger participants. This differs from previous LCA applications in dentistry, where the participants were from narrow age ranges [13]. Although age adjustment was included in the LCA model training to try and reduce this effect, age effects were still highly visible in the LCA groups. This suggests that alternative methods may be needed when strongly age‐patterned traits, such as caries, are evaluated.

The study verified data accuracy on the number of existing or missing teeth and DMFS‐scores from the SKaPa register, covering 50%–75% of the adult population. The SKaPa data showed excellent agreement within a two‐year window with reference assessments made in MODS. This is consistent with previous findings of high concordance in children [17]. Concordance diminished when the interval between SKaPa and the reference data increased, which is anticipated, given that new caries or treatment will occur over time. Using SKaPa data within 2 years of exposure may provide a reasonable balance between sample size and measurement accuracy.

The strength of the present study is the large population‐based derivation cohort and the inclusion of a replication cohort in a distant region of the country with a partly different social context. The main limitation is that SKaPa data does not cover all clinics in Sweden and does not capture people who do not attend a dentist, making it impossible to exclude selection bias.

The study leaves some unanswered questions about the role of these LCA groups in clinical practice and public health, which is suggested as a target for future research. Alternative approaches to interrogate latent structure which account for age better are also needed.

## Author Contributions

I.J. and S.H. initiated and designed the study. N.F., S.H., I.J., L.K., A.E. contributed data analyses and illustrations. D.J. is the co‐initiator and lead designer of MODS. D.J. and P.P. provided and compiled clinical registration data used for the validation section. S.H., I.J. and L.K. drafted the manuscript, and all authors contributed to and approved of the final version.

## Conflicts of Interest

The authors declare no conflicts of interest.

## Supporting information


Appendix S1.
**Figure S1.** Bar and line plots demonstrating decreasing AIC and BIC values across (A) nine LCA classes in VIP and (B) seven classes in MOS. Due to its size, the MOS cohort cannot be divided beyond seven classes. The models include sex and age as covariates.
**Figure S2.** Panel of odontograms illustrating mean caries prevalence per tooth surface for (A) the five LCA classes (class I–V) and (B) the five quintiles based on the DMFS distribution (Q1–Q5) in the MOS cohort. White indicates caries‐free (0) and dark red that all surfaces are caries‐affected (1).
**Table S1.** Baseline dental status and medical phenotype characteristics by (A) LCA class, and (B) quintile ranking in sex and 10‐year age strata in VIP participants. (C) AIC values from LCA or Quintile rank group models, their ratio and *p*‐value for non‐superiority test.
**Table S2.** Dental status and medical phenotype characteristics by (A) LCA class, and (B) quintile ranking in sex and 10‐year age strata in VIP participants with 5‐year follow‐up data. (C) AIC values from LCA or quintile rank group models, their ratio and *p*‐value for non‐superiority test.
**Table S3.** Baseline dental status and medical phenotype characteristics by (A) LCA class, and (B) quintile ranking in sex and 10‐year age strata in MOS participants. (C) AIV values from LCA or Quintile rank group models, their ratio, and *p*‐value for non‐superiority test.
**Table S4.** Dental status and medical phenotype characteristics by (A) LCA class and (B) quintile ranking in MOS participants with 5‐year follow‐up data. (C) AIC values from LCA or quintile rank group models, their ratio and *p*‐value for non‐superiority test.

## Data Availability

The data that support the findings of this study are available on request from the corresponding author. The data are not publicly available due to privacy or ethical restrictions.

## References

[1] A. Bellatorre , S. H. Jackson , and K. Choi , “Development of the Diabetes Typology Model for Discerning Type 2 Diabetes Mellitus With National Survey Data,” PLoS One 12, no. 3 (2017): e0173103. 28253317 10.1371/journal.pone.0173103 PMC5333874

[2] R. G. Verhaak , K. A. Hoadley , E. Purdom , et al., “Integrated Genomic Analysis Identifies Clinically Relevant Subtypes of Glioblastoma Characterized by Abnormalities in PDGFRA, IDH1, EGFR, and NF1,” Cancer Cell 17, no. 1 (2010): 98–110. 20129251 10.1016/j.ccr.2009.12.020 PMC2818769

[3] C. M. Ulbricht , S. A. Chrysanthopoulou , L. Levin , and K. L. Lapane , “The Use of Latent Class Analysis for Identifying Subtypes of Depression: A Systematic Review,” Psychiatry Research 266 (2018): 228–246. 29605104 10.1016/j.psychres.2018.03.003 PMC6345275

[4] N. M. Scheltens , F. Galindo‐Garre , Y. A. Pijnenburg , et al., “The Identification of Cognitive Subtypes in Alzheimer's Disease Dementia Using Latent Class Analysis,” Journal of Neurology, Neurosurgery, and Psychiatry 87, no. 3 (2016): 235–243. 25783437 10.1136/jnnp-2014-309582

[5] L. Hood and S. H. Friend , “Predictive, Personalized, Preventive, Participatory (P4) Cancer Medicine,” Nature Reviews. Clinical Oncology 8, no. 3 (2011): 184–187. 10.1038/nrclinonc.2010.227 21364692

[6] N. J. Kassebaum , A. G. C. Smith , E. Bernabe , et al., “Global, Regional, and National Prevalence, Incidence, and Disability‐Adjusted Life Years for Oral Conditions for 195 Countries, 1990–2015: A Systematic Analysis for the Global Burden of Diseases, Injuries, and Risk Factors,” Journal of Dental Research 96, no. 4 (2017): 380–387. 28792274 10.1177/0022034517693566 PMC5912207

[7] C. J. Moores , S. A. M. Kelly , and P. J. Moynihan , “Systematic Review of the Effect on Caries of Sugars Intake: Ten‐Year Update,” Journal of Dental Research 101, no. 9 (2022): 1034–1045. 35302414 10.1177/00220345221082918

[8] J. L. Rodriguez , M. Thakkar‐Samtani , L. J. Heaton , E. P. Tranby , and T. Tiwari , “Caries Risk and Social Determinants of Health: A Big Data Report,” Journal of the American Dental Association (1939) 154, no. 2 (2023): 113–121. 36503669 10.1016/j.adaj.2022.10.006

[9] J. E. Frencken , P. Sharma , L. Stenhouse , D. Green , D. Laverty , and T. Dietrich , “Global Epidemiology of Dental Caries and Severe Periodontitis–A Comprehensive Review,” Journal of Clinical Periodontology 44, no. Suppl 18 (2017): S94–S105. 28266116 10.1111/jcpe.12677

[10] J. R. Shaffer , E. Feingold , X. Wang , et al., “Clustering Tooth Surfaces Into Biologically Informative Caries Outcomes,” Journal of Dental Research 92, no. 1 (2013): 32–37. 23064960 10.1177/0022034512463241 PMC3521447

[11] S. Haworth , A. Esberg , P. Lif Holgerson , et al., “Heritability of Caries Scores, Trajectories, and Disease Subtypes,” Journal of Dental Research 99, no. 3 (2020): 264–270. 31905308 10.1177/0022034519897910 PMC7036480

[12] M. Simancas‐Pallares , A. Gormley , P. Shrestha , et al., “Evidence for Clinical Subtypes of Early Childhood Caries,” 2023. 10.1111/cdoe.12795 PMC10102252 36239051

[13] A. Gormley , S. Haworth , M. Simancas‐Pallares , et al., “Subtypes of Early Childhood Caries Predict Future Caries Experience,” Community Dentistry and Oral Epidemiology 51, no. 5 (2023): 966–975. 36239051 10.1111/cdoe.12795 PMC10102252

[14] I. von Bultzingslowen , H. Ostholm , L. Gahnberg , D. Ericson , J. L. Wennstrom , and J. Paulander , “Swedish Quality Registry for Caries and Periodontal Diseases–A Framework for Quality Development in Dentistry,” International Dental Journal 69, no. 5 (2019): 361–368. 31001827 10.1111/idj.12481 PMC6790561

[15] M. Norberg , S. Wall , K. Boman , and L. Weinehall , “The Vasterbotten Intervention Programme: Background, Design and Implications,” Global Health Action 3 (2010): 3. 10.3402/gha.v3i0.4643 PMC2844807 20339479

[16] L. Brunkwall , D. Jonsson , U. Ericson , et al., “The Malmo Offspring Study (MOS): Design, Methods and First Results,” European Journal of Epidemiology 36, no. 1 (2021): 103–116. 33222051 10.1007/s10654-020-00695-4 PMC7847466

[17] T. Mensah , S. Tranaeus , A. Cederlund , A. Naimi‐Akbar , and G. Klingberg , “Swedish Quality Registry for Caries and Periodontal Diseases (SKaPa): Validation of Data on Dental Caries in 6‐ and 12‐Year‐Old Children,” BMC Oral Health 21, no. 1 (2021): 373. 34301237 10.1186/s12903-021-01705-x PMC8305535

[18] J. Manjer , S. Carlsson , S. Elmstahl , et al., “The Malmo Diet and Cancer Study: Representativity, Cancer Incidence and Mortality in Participants and Non‐Participants,” European Journal of Cancer Prevention 10, no. 6 (2001): 489–499. 11916347 10.1097/00008469-200112000-00003

[19] I. Johansson , G. Hallmans , A. Wikman , C. Biessy , E. Riboli , and R. Kaaks , “Validation and Calibration of Food‐Frequency Questionnaire Measurements in the Northern Sweden Health and Disease Cohort,” Public Health Nutrition 5, no. 3 (2002): 487–496. 12003662 10.1079/phn2001315

[20] I. Johansson , B. Van Guelpen , J. Hultdin , M. Johansson , G. Hallmans , and P. Stattin , “Validity of Food Frequency Questionnaire Estimated Intakes of Folate and Other B Vitamins in a Region Without Folic Acid Fortification,” European Journal of Clinical Nutrition 64, no. 8 (2010): 905–913. 20502473 10.1038/ejcn.2010.80

[21] L. Wang , Z. Zhang , J. J. McArdle , and T. A. Salthouse , “Investigating Ceiling Effects in Longitudinal Data Analysis,” Multivariate Behavioral Research 43, no. 3 (2009): 476–496. 19924269 10.1080/00273170802285941 PMC2778494

[22] T. M. Nguyen , U. Tonmukayakul , L. K. Le , H. Calache , and C. Mihalopoulos , “Economic Evaluations of Preventive Interventions for Dental Caries and Periodontitis: A Systematic Review,” Applied Health Economics and Health Policy 21, no. 1 (2023): 53–70. 36089630 10.1007/s40258-022-00758-5 PMC9834378

